# Glutathione depletion in survival and apoptotic pathways

**DOI:** 10.3389/fphar.2014.00267

**Published:** 2014-12-04

**Authors:** Milena De Nicola, Lina Ghibelli

**Affiliations:** ^1^Dipartimento di Biologia, Universita' di Roma Tor VergataRoma, Italy; ^2^Dipartimento di Scienze e Tecnologie Chimiche, Universita' di Roma Tor VergataRoma, Italy

**Keywords:** apoptosis, GSH, GSH transporters, BSO, NF-KappaB

## GSH extrusion in apoptosis

Damaged cells opting for apoptosis actively extrude glutathione in the reduced form as an apical event in the apoptotic signaling (Ghibelli et al., 1995; Van den Dobbelsteen et al., 1996). Apoptotic GSH efflux is very rapid and, as a consequence, cells undergo redox imbalance and become susceptible to oxidative stress due to loss of ROS scavenging power (D'Alessio et al., 2003); moreover, the sudden redox disequilibrium causes the formation of inter-protein disulfides among reactive exposed cysteines, altering cell functions and signaling in a pro-apoptotic fashion (Circu and Aw, 2012).

### GSH efflux in the life and death of a cell

Glutathione is synthesized in the cell cytosol, and acts essentially as an intracellular antioxidant. However, viable cells (mainly hepatocytes and macrophages) extrude GSH as part of their physiological functions; this provides antioxidant protection to the extracellular environment (Bachhawat et al., 2013). The passage of GSH through plasma membrane is regulated by a switch in the open/closed configuration of the transporters, and occurs according to gradient, being in fact uni-directional (i.e., export rather than import) because intracellular levels are much higher than those of body fluids. GSH transporters were originally functionally identified as belonging to the sinusoidal or canalicular type according to their position in the hepatic anatomy, and to their responsiveness to specific inhibitors (Yi et al., 1995). However, their molecular nature is still elusive (Bachhawat et al., 2013). Based on several experimental evidences, the prevalent current opinion is that GSH transporters coincide with the multi-drug resistance associated proteins (MRP) (Ballatori et al., 2009; Franco and Cidlowski, 2012), which constitute the complexes responsible for cell detoxification from xenobiotics through the export of glutathione-S conjugates produced by glutathione-S-transferase (Pompella et al., 2003). The regulation of the activity of GSH transporters is still unexplored, possibly being controlled by the differential concentration of GSH in the vicinity of the carriers in the internal vs. external face of the cell membrane, thereby relying in the zonal control of GSH levels by intracellular trafficking.

A different scenario applies to the export of the oxidized glutathione dimers (GSSG) formed during oxidative stress (and not reduced back to GSH by glutathione reductase). Even though GSSG was shown to be target of MRP (Ballatori et al., 2009), in conditions of oxidative stress GSSG exits from cells passively crossing the plasma membrane. This mechanism helps avoiding a dangerous drop in the GSH/GSSG ratio due to the accumulation of GSSG, and the consequent redox imbalance.

Glutathione loss in apoptosis has been subject of intense investigation. Most apoptotic cells become devoid of glutathione even before rupture of the plasma membrane (Ghibelli et al., 1995), which indicates an active phenomenon of extrusion. In instances of apoptosis induced by oxidative insults, a passive efflux of GSSG was observed (Esteve et al., 1999); in this case, GSH loss is a consequence of oxidative stress. In instances of apoptosis induced by non-oxidative insults, glutathione is extruded outside cells in the reduced form immediately prior to the execution of apoptosis (Ghibelli et al., 1995; Van den Dobbelsteen et al., 1996), followed by ROS production as a consequence (D'Alessio et al., 2003); in this case, GSH loss is the cause, rather than the consequence of oxidative stress.

Apoptotic GSH extrusion is prevented by inhibitors of the sinusoidal GSH transporters (Ghibelli et al., 1998), indicating that apoptotic GSH efflux follows the same route as physiological GSH export. However, while healthy cells shed only a limited fraction of their GSH, in apoptosis the efflux leads to complete GSH depletion, suggesting that the transporters remain stably in an open configuration. The events that cause this phenomenon are still unknown; we have preliminary evidence that the activation of the apoptotic protease caspase-2, but not the other caspases, is required for apoptotic GSH efflux, suggesting that a proteolytic event (the digestion of an inhibitor of the transporters?), might cause an irreversible opening of the GSH transporters (De Nicola et al., in preparation).

### Role of apoptotic GSH extrusion

GSH extrusion is a necessary step of the apoptotic signal transduction (Coppola and Ghibelli, 2000), occurring as an apical and causative event, as shown by studies demonstrating that inhibitors of GSH efflux also inhibit apoptosis (Ghibelli et al., 1998). The block of the apoptotic signaling occurs as a very early step, because even apical events such as Bax translocation are prevented. Antioxidants do not hamper apoptotic GSH efflux, but protect GSH-depleted cells from apoptosis (Liuzzi et al., 2003), indicating that the sudden GSH drop activates a redox-sensitive target among the apical pro-apoptotic proteins. A likely target is Bax, a pro-apoptotic member of the Bcl-2 family that in apoptosis moves to mitochondria, where it is responsible for the release of cytochrome c and the consequent caspase activation and cell demise (Ghibelli and Diederich, 2010). Bax has two exposed cysteines (Suzuki et al., 2000) able to form inter-protein disulfide bridges upon oxidation (D'Alessio et al., 2005), and therefore it can be at the same time a sensor of oxidation and an effector of apoptosis. Indeed, Bax was experimentally shown to assemble in an oxidative dimer configuration, putatively forming a disulfide between cysteine 62 and cysteine 126, thereby acquiring the ability to translocate to mitochondria (D'Alessio et al., 2005) and promote cytochrome c release (Ghibelli et al., 1999).

Damage-induced apoptosis was shown to follow either redox-sensitive or -insensitive routes, characterized by GSH depletion or retention, respectively, through alternative biochemical routes that can be morphologically identified (De Nicola et al., 2006). Leukemic cells display multiple nuclear apoptotic morphologies; in particular, two types of nuclear vesiculation (budding and cleavage) are the result of two independent morphological routes, since they never interconvert and are differently modulated by damage vs. physiological apoptogenic agents (Dini et al., 1996). *In situ* analyses showed that intracellular GSH is completely extruded in cells undergoing apoptosis by cleavage, whereas it is fully maintained in apoptotic budding cells (De Nicola et al., 2006). Accordingly, *in situ* measurement of ROS levels showed that only cells in cleavage develop oxidative stress (Celardo et al., 2011). GSH depletion, and the consequent oxidation, is not only concomitant to, but is the determinant of the cleavage route, since inhibition of the sinusoidal GSH transporters completely prevents the appearance of the apoptotic cells displaying the budding morphology (De Nicola et al., 2006).

## Pro-survival pathways induced by GSH depletion

GSH, as a co-factor of the glutathione-S-transferase set of enzymes, plays a major role in the detoxification of cells from exogenous molecules, including pharmaceutical drugs. The depletion of GSH, achieved in clinics with the specific GSH synthesis inhibitor buthionine sulphoxymine (BSO), is an important strategy to improve drug efficacy in anticancer therapies, since many aggressive tumor cells are very efficient in drug extrusion (multi-drug resistant, MDR), thereby nullifying the effects of chemotherapies. In these cases BSO acts a chemosensitizer, since GSH depletion allows bypassing the MDR obstacle, achieving efficacious levels of antitumor drug within MDR cells (Fojo and Bates, 2003).

BSO is a specific inhibitor of glutathione synthase, thereby in BSO-treated cells the GSH pool is consumed but not replaced, and its level declines according to the consumption rate, with a kinetics that depends on the oxidative status of the cells. In any case, the depletion is much slower compared with what occurs during apoptosis (hours vs. minutes). This slow rate gives time to the BSO-treated cells to adapt to the progressively oxidizing environment, setting up stress response pathways aimed at increasing cell survival (Rahman et al., 2005; Ishii and Mann, 2014). Such responses can be very robust, and in non-MDR tumor cells BSO, rather than acting as a chemo-sensitizer, paradoxically potentiates cell survival, thereby decreasing the pro-apoptotic efficiency of anti-tumor drugs (D'Alessio et al., 2004).

The survival responses to GSH depletion include the transcriptional activation of genes encoding for cell protective proteins, *via* the nuclear factor kappaB (NF-kappaB) (Filomeni et al., 2005) or the NF-E2-related factor 2 (Nrf-2) (Lee et al., 2008) transcriptional activators.

NF-kappaB is a transcription complex that in response to many insults, including oxidative stress, mediates the transactivation of key cell protective proteins (Morgan and Liu, 2011). BSO-mediated activation of NF-kappaB leads to multiple cell-protective responses, such as the trans-activation of heat shock protein 27, which stops the apoptotic signal after Bax translocation and cytochrome c release (Filomeni et al., 2005), and the main anti-apoptotic protein Bcl-2. Bcl-2 is over-expressed in BSO-treated cells via transcriptional activation, both in tumor cells (D'Alessio et al., 2004) and primary lymphocytes (Cristofanon et al., 2006) and monocytes (Cristofanon et al., 2008). The increased Bcl-2 synthesis compensates for, and overcome, the faster catabolism of the Bcl-2 protein occurring in cells with low GSH levels (Meredith et al., 1998; D'Alessio et al., 2004), increasing the intracellular Bcl-2 levels up to three-four folds (D'Alessio et al., 2004). This is achieved through the activation of the so-called non-canonical NF-kappaB pathway by a two-step process. First, as a fast response to GSH depletion, a ternary disulfide complex forms between two subunits of the NF-kappaB component p50 and its tutoring protein Bcl-3 (Cristofanon et al., 2009). Second, the complex is then activated *via* a mechanism dependent on the mitogen-activated protein p38, a kinase that requires high ROS levels (Filomeni et al., 2003); this occurs as a consequence of GSH depletion only several hours later (Limón-Pacheco et al., 2007; Cristofanon et al., 2009).

Nrf-2 is a transcription factor activated by redox imbalance via the removal of its redox-sensitive anchor Keap1 (Kundu and Surh, 2010), and is devoted to the transcription of genes encoding for glutathione metabolism and antioxidant enzymes (Li and Kong, 2009; Ramprasath and Selvam, 2013), thus providing cells with extra antioxidant defense, and reducing the apoptotic outcome. It was found that murine embryonic fibroblasts survive to BSO treatment due to a strong Nrf-2 response, whereas Nrf-2 deficient cells, or cells treated with Nrf2 siRNA became sensitive to BSO-induced apoptosis (Lee et al., 2008).

Therefore, the straightforward use of BSO as coadjuvant in anticancer therapies should be limited to tumors bearing MDR cells. Importantly, non-MDR cells could develop chemo-resistance as a result of BSO treatment, unless they are targeted with specific RNA interfering agents to prevent the over-expression of survival proteins.

## Conclusions

**Figure 1:**
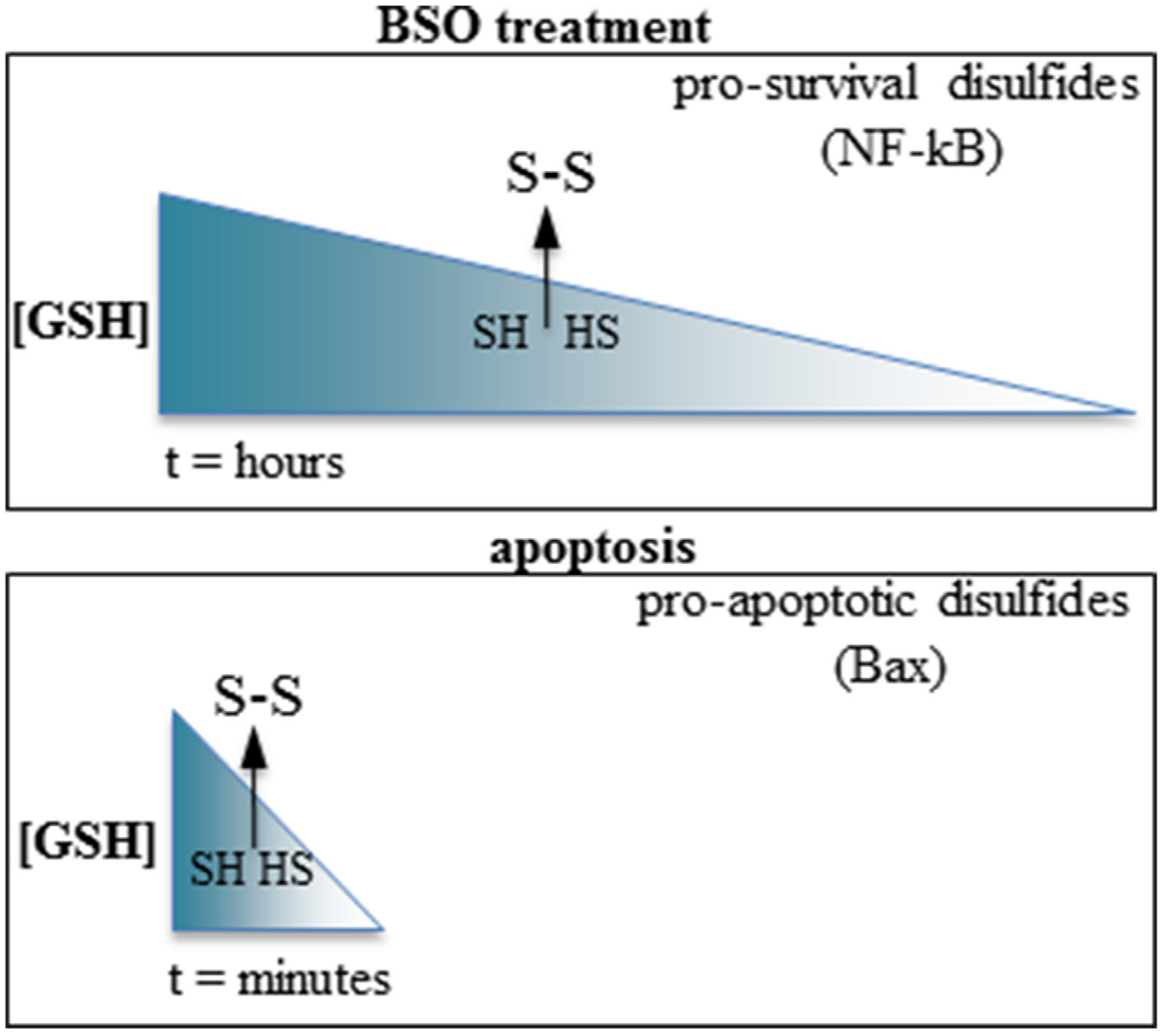
**Slow vs. fast GSH depletion produce opposite effects on cell survival. Top:** BSO induce slow GSH depletion (hours); the disulfides formed after reaching a threshold level of redox imbalance possess a cell-survival activity (e.g., the p50 subunit of NF-kappaB). **Bottom:** the very rapid GSH extrusion occurring in apoptosis promote the formation of pro-apoptotic disulfides (e.g., Bax). The arrows indicate a hypothetical threshold level of GSH depletion after which sulfhydryls (SH, HS) are oxidized to disulfides (S-S) in each condition.

Summarizing, GSH depletion may promote either pro-apoptotic or pro-survival pathways depending on the kinetic of depletion; swift GSH extrusion activates pro-apoptotic pathways, whereas slower GSH depletion activates pro-survival pathways (Figure 1). Intriguingly, both types of response require increased ROS levels and formation of inter-protein disulfides to be activated. The reactivity of cysteines depends on the surrounding aminoacids determining protein conformation, and each inter-protein cystine forms at fixed levels of oxidation of the cell microenvironment. It is conceivable that the reactive cysteines of the proteins involved in the opposite responses (e.g., the cell-protective NF-kappaB component p50 vs. the pro-apoptotic Bax) may be differently modulated according to the swiftness of the redox imbalance, possibly differently relying in the tutoring effect of thiol-modifying enzymes such as thioredoxin or protein disulfide isomerase.

### Conflict of interest statement

The authors declare that the research was conducted in the absence of any commercial or financial relationships that could be construed as a potential conflict of interest.
